# Bisphosphonate‐enoxacin inhibit osteoclast formation and function by abrogating RANKL‐induced JNK signalling pathways during osteoporosis treatment

**DOI:** 10.1111/jcmm.16949

**Published:** 2021-10-15

**Authors:** Qiang Xu, Ping Zhan, Xiaofeng Li, Fengbo Mo, Huaen Xu, Yuan Liu, Qi Lai, Bin Zhang, Min Dai, Xuqiang Liu

**Affiliations:** ^1^ Department of Orthopedics The First Affiliated Hospital of Nanchang University Artificial Joints Engineering and Technology Research Center of Jiangxi Province Nanchang China

**Keywords:** bisphosphonate‐enoxacin, bone‐targeting, osteoclast, osteoporosis

## Abstract

Osteoporosis is an age‐related disease characterized by low mineral density, compromised bone strength and increased risk of fragility fracture. Most agents for treating osteoporosis focus primarily on anti‐resorption by inhibiting osteoclast activity. Bisphosphonate (BP) is a potent anti‐resorptive agent that has been used clinically for decades and is proven to be effective. However, BP has a variety of side effects and is far from being an ideal anti‐osteoporosis agent. BP selectively binds to calcium crystals, which are subsequently taken up or released by osteoclasts. Based on the action of BP, we previously demonstrated the inhibitory effect of a novel bone‐targeting BP derivative, bisphosphonate‐enoxacin (BE). In the current study, we used bone marrow‐derived osteoclast cultures to further assess the inhibitory effect of BE on osteoclastogenesis and employed reverse transcription PCR and real‐time PCR to examine expression of osteoclast‐specific genes. Additionally, we used bone resorption and F‐actin immunofluorescence assays to evaluate the effect of BE on osteoclast function and investigated the potential mechanisms affecting osteoclast differentiation and function *in vitro*. Furthermore, an ovariectomized (OVX) rat model was established to evaluate the therapeutic effects of BE on preventing bone loss. Results showed that BE exerted potent inhibitory effects on osteoclast formation and bone resorption by specifically abrogating RANKL‐induced JNK signalling, and that it preserved OVX rat bone mass *in vivo* without any notable side effects. Collectively, these results indicated that the BP derivative BE may have significant potential as a treatment for osteoporosis and other osteolytic diseases.

## 1 INTRODUCTION

Bone homeostasis is maintained through two coordinated actions, osteoblast bone formation and osteoclast bone resorption, which is a process called ‘bone remodelling’.^1^ If the balance is disrupted, there will be an accordingly increase or decrease in bone mass. Excess osteoclast formation or activation is responsible for most osteolytic diseases, including osteoporosis, rheumatoid arthritis and aseptic osteolysis following total joint replacement.^2^, ^3^, ^4^, ^5^ Thus, osteoclasts are considered key targets in treating these diseases. Identification of agents that can modulate aberrant osteoclast formation and resorptive function is a viable strategy in the development of bone‐protective therapies.

As first reported by Fleisch, bisphosphonates (BP) have high affinity for calcium crystals, leading to the impairment of their formation and dissolution *in vivo* and thereby inhibiting bone resorption.^6^, ^7^, ^8^ As a result of this activity, BP has been used clinically over the past two decades as a treatment for osteoporosis and has been proven effective in improving bone strength and decreasing the risk of fracture.^9^, ^10^, ^11^ However, other research has indicated that BP may be incorporated into bone during prolonged treatment, resulting in bone metabolism being affected for extended periods, even after the cessation of BP intake.^12^, ^13^ Although the deposition of BP in bone tissue is considered relatively safe, events such as gastrointestinal discomfort, atypical femora fracture and osteonecrosis of the jaw do occur.^14^, ^15^, ^16^, ^17^ Given these adverse side effects, the use of BP is somewhat limited and there is a clinical need to identify novel anti‐resorptive agents that can be developed for treating osteoporosis and other osteolytic diseases.

Enoxacin, a fluoroquinolone antibiotic, was reported to have the properties of inhibiting osteoclast formation and function by interfering the interaction between V‐ATPase B2‐subunit and microfilaments.^18^, ^19^ Furthermore, we previously reported that enoxacin has an inhibitory effect on osteoclastogenesis and reduces titanium‐particle‐induced osteolysis through suppression of the JNK signalling pathway.^20^ It is intriguing that this widely used antibiotic has these unexpected properties, which may translate into its clinical use for preventing osteoporosis and other osteoclast‐related bone diseases. However, it is of concern that patients undergoing long‐term treatment with enoxacin may experience adverse effects, such as dysbacteriosis and gastrointestinal discomfort. To overcome these potential difficulties, we developed a BP derivative of enoxacin (BE), aiming to target bone with the high affinity of BP to calcium crystals and thereby preventing the side effects associated with other systems.

Previous research has shown that BE has a significant inhibitory effect on osteoclast formation *in vitro* and that it is beneficial for preventing orthodontic tooth movement and alveolar bone resorption *in vivo*.^21^, ^22^ However, the potential mechanisms regarding the inhibitory effect of BE on osteoclastogenesis remain to be investigated. Furthermore, the efficacy of BE on preventing ovariectomized (OVX)‐induced bone loss and the safety of this novel agent are currently unclear. Therefore, in the current study, we investigated the direct effect of BE on osteoclast formation and resorption function *in vitro* and compared it with zoledronate, a widely used nitrogen‐containing bisphosphonate. We also explored the molecular mechanisms of BE activity and established an OVX rat model to evaluate this novel agent *in vivo* with respect to its safety, therapeutic effects on preserving bone mass, and effect on bone turnover. We found that BE functioned differently than BP and provided equal or better therapeutic efficacy, both *in vitro* and *in vivo*. Taken together, we believe that the use of BP derivatives may be a viable approach towards reducing the occurrence of side effects associated with long‐term BP treatment and may reduce the occurrence of unsatisfactory situations when treating osteoporosis and other osteoclast‐related bone destructive diseases.

## 2 MATERIALS AND METHODS

### 2.1 Media and reagents

RAW264.7 cells were purchased from the American Type Culture Collection (ATCC). Alpha modification of Eagle medium (α‐MEM) and foetal bovine serum (FBS) were purchased from Gibco‐BRL. A cell counting kit (CCK‐8) was obtained from Dojindo. Recombinant soluble human macrophage colony‐stimulating factor (M‐CSF) and mouse receptor activator of nuclear factor‐κB ligand (RANKL) were obtained from R&D Systems. Bisphosphonate‐enoxacin (BE) was purchased from SynQuest Laboratory (Product #8H77‐B‐06). Specific antibodies against ERK, JNK, p38, IκBα, phospho‐ERK (Thr202/Tyr204), phospho‐JNK (Thr183/Tyr185), phospho‐p38 (Thr180/Tyr182), phospho‐IκBα (Ser32) and GAPDH were obtained from Cell Signaling Technology. The tartrate‐resistant acid phosphatase (TRAP) staining kit (Diagnostic Acid Phosphatase Kit) and all other reagents were purchased from Sigma Aldrich, unless stated otherwise.

### 2.2 Cell culture and osteoclast differentiation assays

We harvested bone marrow monocyte/macrophage cells (BMMs) from four to six‐week‐old C57BL/6 mice, as previously described.^23^ Briefly, cells were isolated from the femur and tibiae bone marrow and cultured for 24 h in T75 flasks containing α‐MEM supplemented with 10% FBS, 1% penicillin/streptomycin and 10 ng/mL M‐CSF. Non‐adherent cells were removed, and the adherent cells were cultured in a 37 °C, 5% CO_2_ incubator for an additional 3–4 days. When the BMMs were 90% confluent, they were washed three times with phosphate‐buffered saline (PBS) and trypsinised. We then seeded the cells into 96‐well plates at a density of 8 × 10^3^ cells/well^24^ in complete α‐MEM supplemented with 30 ng/ml M‐CSF, 50 ng/ml RANKL, 2.5 μM zoledronate (ZOL) or various concentrations of BE (0, 5 or 10 μM). The cell culture media were replaced every 2 days until mature osteoclasts formed. The cells were then washed twice with PBS, fixed with 4% paraformaldehyde for 20 min and stained for TRAP using the Diagnostic Acid Phosphatase Kit. The cells were evaluated by light microscopy, and TRAP‐positive cells with more than three nuclei were counted.

### 2.3 Cell viability assays

To assess the cytotoxicity of ZOL and BE on BMMs, we performed CCK‐8 assays. According to the manufacturer's instructions, BMMs were plated into 96‐well plates at a density of 8 x 10^3^ cells/well and cultured in complete α‐MEM supplemented with 30 ng/mL M‐CSF. After 24 h, the cells were treated with different concentrations of ZOL and BE for 2 days. Next, 10 μl of CCK‐8 buffer was added to each well and incubated at 37°C, 5% CO2 for an additional 2 h. We then measured absorbance at a wavelength of 450 nm (650 nm reference) using an ELX800 Absorbance Microplate Reader (Bio‐Tek). The following formula was used to calculate cell viability compared to control cells: (experimental group OD – zeroing OD)/(control group OD – zeroing OD).

### 2.4 Resorption pit and F‐actin ring immunofluorescence

We seeded BMMs onto bovine bone slices in 96‐well plates at a density of 8 x 10^3^ cells/well. The cells were then induced every other day with complete α‐MEM supplemented with 30 ng/ml M‐CSF and 50 ng/ml RANKL. Once mature osteoclast were observed, cells were subsequently treated with ZOL (2.5 μM) and different concentrations of BE (0, 5 and 10 μM) for another 48 h. Cells treated with PBS were used as negative controls. Adherent cells on the bone slices were removed, and resorption pits were visualized using an FEI Quanta 250 scanning electron microscope (SEM). The percentage of absorbance area was quantified for the bone slices using Image J software (National Institutes of Health). In addition, we observed F‐actin ring (Ruffled membrane of osteoclast) formation using immunofluorescence assays as a more in depth investigation of osteoclast bone resorption function. For this, BMMs were cultured and treated with ZOL, varying concentrations of BE on bovine bone slices as the same way. The mature osteoclasts were then subjected to fixation with 4% paraformaldehyde for 20 min, permeabilization with 0.1% (v/v) Triton X‐100 (Sigma Aldrich) for 5 min, and incubated with Alexa‐Fluor 647 phalloidin (Invitrogen) for 1 h. We then washed the cells three times with PBS, and the phalloidin‐stained cells were then incubated with Hoechst 3342 dye (1:5000; Invitrogen) to stain the nuclei. The cells were washed with PBS and finally mounted with using ProLong Gold Anti‐fade Mountant (Invitrogen). Fluorescent staining of F‐actin ring was evaluated using a NIKON A1Si Spectral Detector Confocal System equipped with 10×(dry) lenses.

### 2.5 Reverse transcription and quantitative PCR

We seeded BMMs into 6‐well plates at a density of 8 x 10^4^ cells/well and cultured them in complete α‐MEM supplemented with 30 ng/ml M‐CSF and 50 ng/ml RANKL. The media were changed every other day. The cells were then treated with 5 or 10 μM BE until mature osteoclasts formed. We isolated total RNA mature osteoclasts using a Qiagen RNeasy Mini Kit (Qiagen) according to the manufacturer's instructions We then synthesized complementary DNA (cDNA) using reverse transcriptase (TaKaRa Biotechnology) and 1 μg of total RNA as template. The cDNA was PCR amplified, and the PCR products electrophoresed on 1% agarose gels. The images were captured using an ImageQuant LAS 4000 Documentation System (GE Healthcare). In addition, real‐time quantitative PCR was performed using an SYBR Premix Ex Tag Kit (TaKaRa) and ABI 7500 Sequencing Detection System (Applied Biosystems). The following PCR amplification parameters were used: 40 cycles of 5 s denaturation at 95°C and 34 s amplification at 60°C. All reactions were performed in triplicate and normalised to the housekeeping gene glyceraldehyde 3‐phosphate dehydrogenase (GAPDH). The primer sequences of mouse osteoclast‐specific genes CathepsinK, CTR, TRAP, dendritic cell‐specific transmembrane protein (DC‐STAMP), V‐ATPase a3, V‐ATPase d2, c‐fos, and NFATc1 and GAPDH were as follows: CathepsinK forward 5′‐CTTCCAATACGTGCAGCAGA‐3′ and CathepsinK reverse 5′‐TCTTCAGGGCTTTCTCGTTC‐3′; CTR forward 5′‐TGCAGACAACTCTTGGTTGG‐3′ and CTR reverse 5′‐TCGGTTTCTTCTCCTCTGGA‐3′; TRAP forward 5′‐CTGGAGTGCACGATGCCAGCGACA‐3′ and TRAP reverse 5′‐TCCGTGCTCGGCGATGGACCAGA‐3′; DC‐STAMP forward 5′‐AAAACCCTTGGGCTGTTCTT‐3′ and DC‐STAMP reverse 5′‐AATCATGGACGACTCCTTGG‐3′; V‐ATPase a3 forward 5′‐ GCCTCAGGGGAAGGCCAGATCG‐3′ and V‐ATPase a3 reverse 5′‐ GGCCACCTCTTCACTCCGGAA‐3′; V‐ATPase d2 forward 5′‐AAGCCTTTGTTTGACGCTGT‐3′ and V‐ATPase d2 reverse 5′‐TTCGATGCCTCTGTGAGATG‐3′; c‐Fos forward 5′‐CCAGTCAAGAGCATCAGCAA‐3′ and c‐Fos reverse 5′‐AAGTAGTGCAGCCCGGAGTA‐3′; NFATc1 forward 5′‐CCGTTGCTTCCAGAAAATAACA‐3′ and NFATc1 reverse 5′‐TGTGGGATGTGAACTCGGAA‐3′; and GAPDH forward 5′‐ACCCAGAAGACTGTGGATGG‐3′ and GAPDH reverse 5′‐CACATTGGGGGTAGGAACAC‐3′.

### 2.6 Western blot analysis

To evaluate protein expression, we seeded RAW264.7 cells into 6‐well plates at a density of 5 × 10^5^ cells/well. Once confluent, we pre‐treated the cells with or without 10 μM BE for 4 h. The cells were then stimulated with 50 ng/ml RANKL for 0, 5, 10, 20, 30 or 60 min. Total protein was extracted from the cultured cells using RIPA lysis buffer containing 50 mM Tris‐HCl, 150 mM NaCl, 5 mM ethylenediaminetetraacetic acid (EDTA), 1% Triton X‐100, 1 mM sodium fluoride, 1 mM sodium vanadate, 1% deoxycholate and a protease inhibitor cocktail. The lysates were centrifuged at 12,000 *g* for 15 min, and the supernatants collected. Protein concentrations were determined using a bicinchoninic acid (BCA) assay. Thirty micrograms of each protein lysate was then resolved by sodium dodecyl sulphate‐polyacrylamide gel electrophoresis (SDS‐PAGE) using 10% gels and transferred to polyvinylidene difluoride membranes (Millipore). After transferring, the membranes were blocked for 1 h with 5% skimmed milk in TBS‐Tween buffer (TBS; 0.05 M Tris, 0.15 M NaCl pH 7.5, and 0.2% Tween‐20). The membranes were then incubated overnight at 4 ℃ with primary antibodies diluted in TBS‐Tween buffer containing 1% (w/v) skimmed milk powder. We then washed the membranes and incubated them with the appropriate secondary antibodies conjugated with IRDye 800CW (molecular weight 1162 Da). Antibody reactivity was detected by exposure using an Odyssey Infrared Imaging System (LI‐COR).

### 2.7 OVX rat model

To analyse the protective effects of BE on osteoporotic bone, we established an OVX rat model as previously described.^25^, ^26^ Briefly, 60 6‐month‐old female Sprague‐Dawley (SD) rats were randomly divided into five groups (*n* = 12/group), including a sham‐surgery group (Sham), mock drug‐treated OVX rat group (Vehicle), OVX rat treated with 50 μg/kg ZOL group (ZOL), low‐dose (5 mg/kg) BE‐treated OVX group (LBE) and high‐dose (10 mg/kg) BE‐treated group (HBE). The ovaries were removed using the following procedures: the rats were anesthetization with 10% chloralhydrate and retroperitoneal incisions made ventral to the rector spinae muscles, just caudal to the last rib; the ovary and associated fat were located and exteriorized by gentle retraction; the ovary was removed and a 3–0 ligatures placed around the cranial portion of the ovary and ovary vessels; finally, the skin incision was closed with one or two non‐absorbable sutures. Rats in drug‐treated groups were intraperitoneally injected with 50 μg/kg ZOL and one of two different concentrations of BE (5 or 10 mg/kg), every other day. The Sham and Vehicle groups were injected with 0.9% sodium chloride every other day. Four weeks after the initiation of treatment, the first cohort of rats (six rats randomly selected from each group) was euthanized with an overdose of sodium pentobarbital (35 mg/kg; Sigma). Femurs, hearts, livers and kidneys were harvested and placed in 4% formalin for 24 h, then transferred to 70% ethanol. Serum was also collected at the time of euthanasia for biochemical and ELISA testing. The remaining rats (second cohort) were continued to be injected on schedule for another 4 week and then euthanized and bone samples harvested as before. Four and 11 days prior to euthanasia of the animals, Calcein green (20 mg/kg) and Alizarin Red S (30 mg/kg) were intraperitoneally injected for double labelling of bone formation surfaces. All procedures associated with the animal experiments were performed in accordance with the Research Ethics Committee of the First Affiliated Hospital of Nanchang University.

### 2.8 Evaluation of bone turnover markers

We analysed tartrate‐resistant acid phosphatase (TRAP5b) and procollagen type I amino‐terminal propeptide (PINP) and as bone turnover markers. TRAP5b is secreted by osteoclasts, and its levels are proportional to the number of osteoclasts. Therefore, TRAP5b is considered a reliable and sensitive marker of bone resorption.^27^ Meanwhile, PINP is produced by osteoblasts and correlates with bone formation and can be used as a specific bone formation marker in postmenopausal osteoporosis.^28^ In the current study, TRAP5b and PINP were quantified using ELISA assays (Rat MidTM Osteocalcin ELISA Kit and Rat TRAPTM Assay Kit, respectively; IDS Inc., Fountain Hills, AZ, USA), according to the manufacturers' instructions.

### 2.9 Micro‐computed tomography (micro‐CT) scanning

Right femurs were fixed and scanned at a voxel size of 10 μm using a high‐resolution Micro‐CT (μCT80, Scanco Medical). Briefly, a total of 400 slices (4 mm/slice) were scanned at a region of the distal femurs beginning at the growth plate as indicated from the scout view and extending proximally along the femur diaphysis. A threshold value 220 was set when constructing the three‐dimensional (3D) images of the original trabecular bones. After 3D construction, a region of interest (ROI) being 200 slices starting 0.3 mm from the most proximal aspect of the growth plate at the distal femur was selected for trabecular bone analysis. To investigate the trabecular structure, bone volume/tissue volume (BV/TV, %), trabecular number (Tb.N, 1/mm), trabecular thickness (Tb. Th, mm) and the trabecular separation (Tb. Sp, mm) were determined.

### 2.10 Histomorphometric analysis

After micro‐CT scanning, the distal right femurs were paraformaldehyde fixed, decalcified in EDTA buffer and paraffin embedded. Sections (5 mm) were subjected to haematoxylin and eosin (H&E) staining and osteoclast‐specific TRAP staining. The sections were photographed using a high‐resolution microscope. In addition, an area 1 mm in height and width, 0.5 mm below the growth plate and excluding the cortical bone, was selected for quantification and statistical analysis using BioQuant software (BioQuant Image Analysis Corporation). The volumes of bone, tissue and TRAP‐positive multinucleated osteoclasts were quantified in all the samples. The other organs, including the hearts, livers and kidneys, were also sectioned and subjected to H&E staining as described for the femur samples.

### 2.11 Bone formation analysis

We also harvest the left femurs when the animals were euthanized and dehydrated them for a week in graded concentrations of ethanol (70%–100%). We then embedded the specimens in polymethyl methacrylate (PMMA). Sagittal sections of the distal femurs were prepared and ground to approximately 30 μM thickness and then examined using a confocal fluorescence microscope for the presence of Alizarin Red S and Calcein green, which marked bone formation surfaces. The mineral apposition rate (MAR) was analysed using Image J software.

### 2.12 Statistical analysis

All the data were expressed as means ± standard deviations (SD). Data analysis was conducted using one‐way analysis of variance (ANOVA). Pairwise comparisons were conducted using Student Neuman‐Keuls (SNK) post hoc test to determine differences. Statistically significant differences were considered as *p* < 0.05.

## 3 RESULTS

### 3.1 BE inhibited RANKL‐induced osteoclastogenesis in vitro without cytotoxicity

**FIGURE 1:**
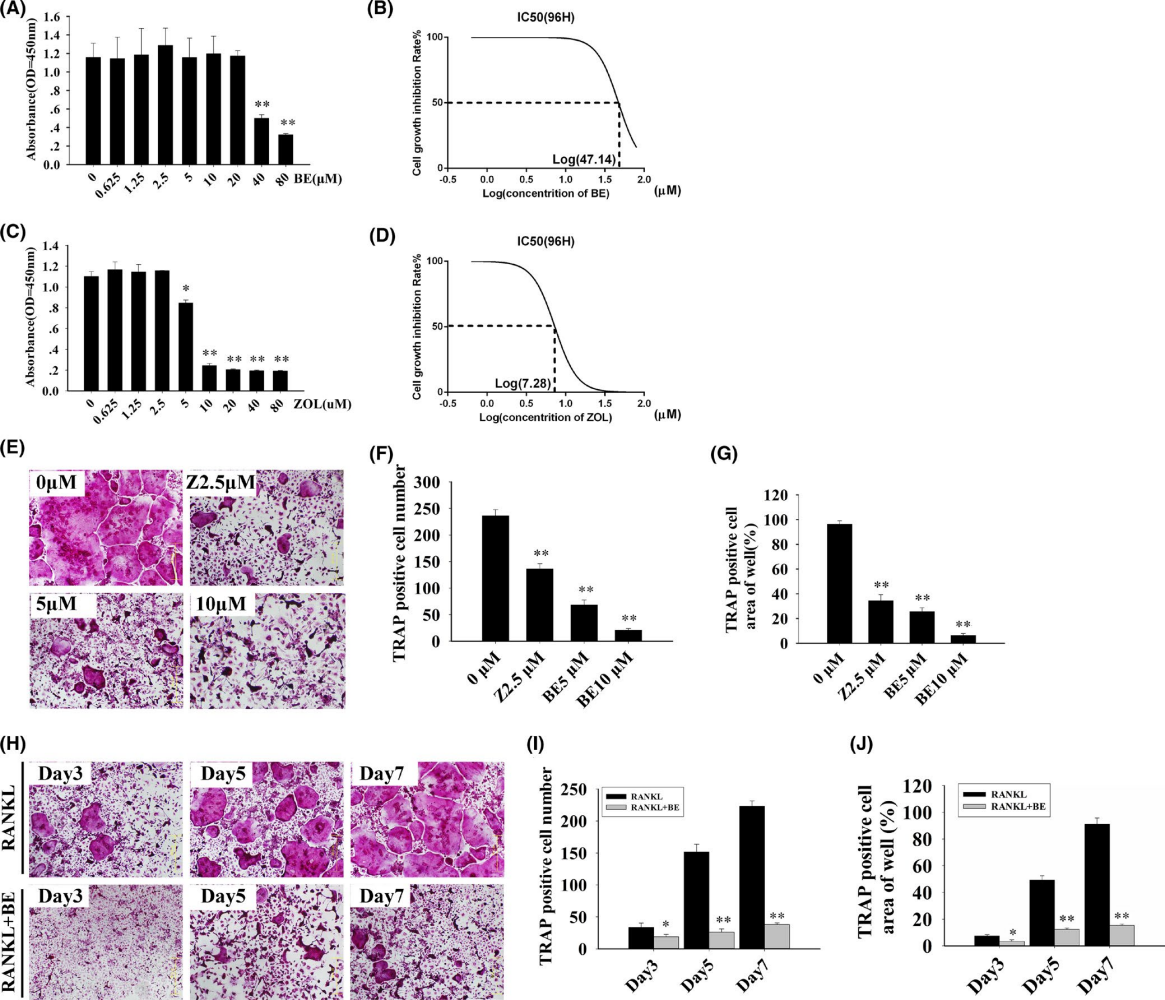
Bisphosphonate‐enoxacin (BE) inhibits RANKL‐induced osteoclastogenesis *in vitro* in dose and time dependent manners without cytotoxicity. (A, C) Cell viability was analysed using a Cell Counting Kit‐8 assay with various concentrations of BE and zoledronate (ZOL) for 96 h. (B, D) The half maximal inhibitory concentration (IC50) of BE and ZOL in bone marrow monocyte/macrophage cells (BMMs) was analysed at 96 h. (E–G) BMMs were cultured in medium containing ZOL (2.5 μM) and various concentrations of BE (0, 5 and 10 μM) for 5–7 d and the numbers and area percentages of tartrate‐resistant acid phosphatase (TRAP)‐positive multinucleated osteoclasts were determined. (H–J) BMMs were incubated in medium with or without 10 μM BE and separately stained for TRAP on Days 3, 5 and 7. All experiments were carried out at least three times. These data are expressed as mean ± standard deviation (SD). *p < 0.05 and **p < 0.01 vs. the control group

First, we used CCK‐8 cell viability/cytotoxicity assays to examine the effect of BE and ZOL on BMM viability. M‐CSF‐dependent BMMs were treated for 96 h with different concentrations of BE and ZOL. As shown in Figure 1A,C, BE and ZOL had no effect on the viability of BMMs at concentrations of ≤20 μM and ≤2.5 μM, respectively. Further analysis of the data allowed us to determine the half maximal inhibitory concentration (IC50) of BE and ZOL for BMMs were 47.14 and 7.28 mg/ml, respectively (Figure 1B,D). Based on these findings, we used 10 μM as the maximum concentration of BE and 2.5 μM as the maximum concentration of ZOL for the subsequent experiments.

To investigate the effects of BE and ZOL on osteoclastogenesis, BMMs were cultured and stimulated with M‐CSF (30 ng/ml) and RANKL (50 ng/ml) and then treated for 5–7 d with ZOL (2.5 μM) or different concentrations of BE (0, 5 and 10 μM). Numerous TRAP‐positive multinucleated osteoclasts were observed in the control group, but the numbers of mature osteoclasts in the BE‐treated and ZOL‐treated groups were significantly reduced, which suggested that both BE and ZOL had inhibitory effects on osteoclast formation (Figure 1E–G). Quantitative analysis indicated that approximately 75 mature osteoclasts/well were observed in the 5 μM BE‐treated group and 140 mature osteoclasts/well were observed in the ZOL‐treated group. In contrast, almost no mature osteoclasts formed in the 10 μM BE‐treated group. Of interest is that TRAP‐positive multinucleated osteoclasts were hardly observed in the 10 μM BE‐treated group compared with that in the ZOL‐treated group. Additionally, analysis of the TRAP‐positive cell area revealed comparable results. To determine the stage that BE exerted its inhibitory affect, BMMs stimulated with RANKL were treated with BE during the early, middle and late stages of osteoclastogenesis. We treated BMMs with 0 or 10 μM BE at 3, 5 and 7 d of development. BE clearly inhibited osteoclast formation at all stages (Figure 1H–J). This suggested that the inhibition of osteoclast differentiation by BE may be both dose and time dependent.

### 3.2 BE attenuated osteoclastic bone resorption and F‐actin ring formation

**FIGURE 2:**
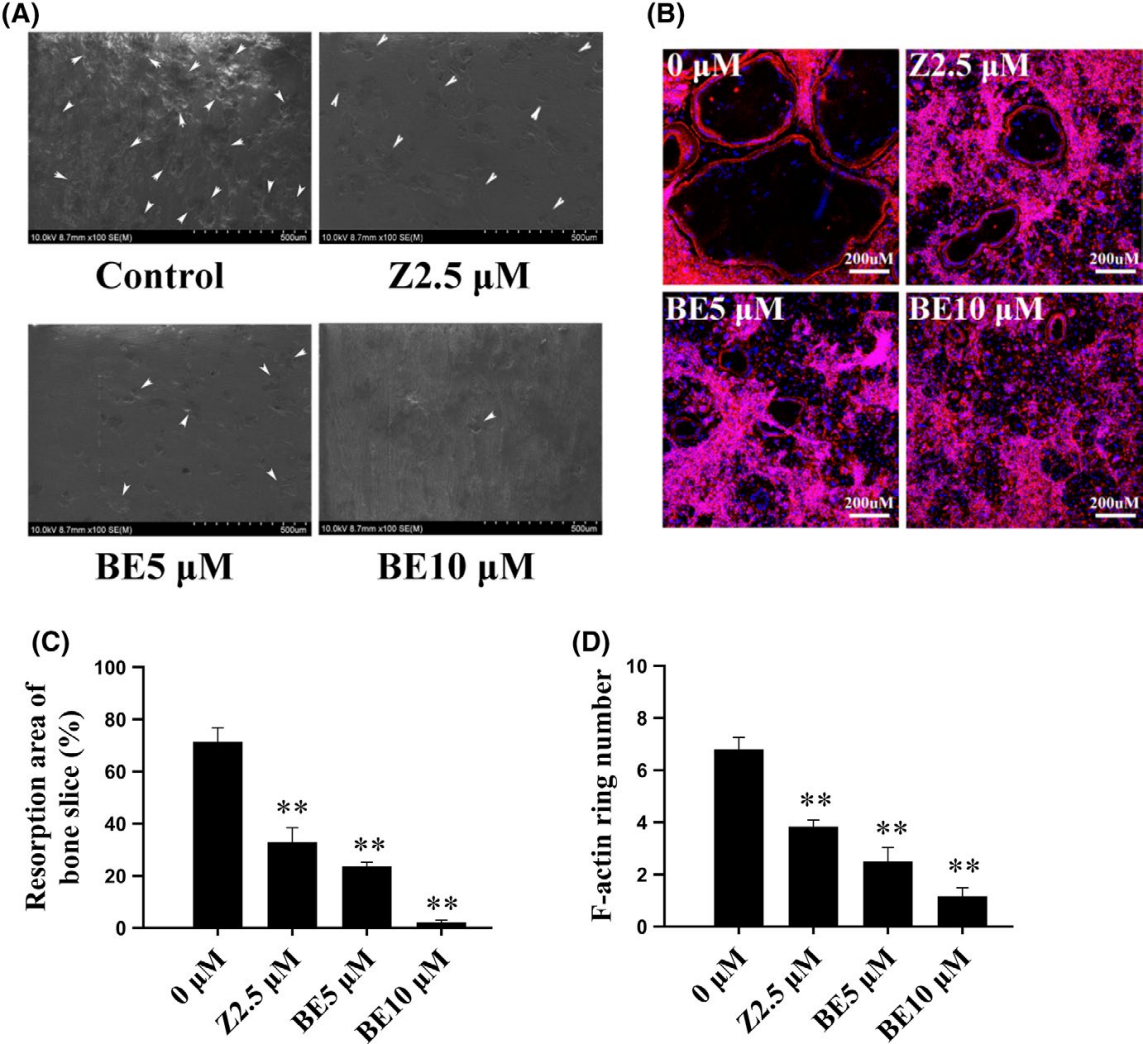
Bisphosphonate‐enoxacin (BE) inhibits osteoclast bone resorption and impairs F‐actin ring formation. (A) Bone marrow macrophages were incubated on bovine bone slices and treated with M‐CSF (30 ng/ml) and RANKL (50 ng/ml), followed by treatment with zoledronate (ZOL; 2.5 μM) and various concentrations of BE (0, 5 and 10 μM) for an additional 48 h. Representative bone resorption observed in scanning electron microscope (SEM) analysis of bone slices. The white arrow indicates bone resorption pits. (B) Cells were then fixed and stained for F‐actin ring formation. (C) Percentage of resorption areas were quantified using Image J software. (D) Numbers of F‐actin ring were quantified. All the experiments were carried out at least three times. The data are expressed as mean ± SD. *p < 0.05 and **p < 0.01 vs. the control group

To determine whether BE impaired the function of osteoclasts, we evaluated the effects of BE on bone resorption and F‐actin ring formation *in vitro*. In untreated control cells, osteoclasts differentiated normally and formed many resorption pits on the bone slices. In contrast, the number of bone resorption pits in the BE‐treated and ZOL‐treated groups was significantly decreased, indicating that both BE and ZOL could inhibited the bone resorption function of osteoclasts (Figure 2A,C). Moreover, the BE‐treated group demonstrated a greater decrease in bone resorption pits compared with that of the ZOL‐treated group. We hypothesized that the reduction of bone resorption pits may have been due to altered F‐actin ring formation. Therefore, we investigated the effect of BE on F‐actin ring formation. BMM‐derived osteoclasts were cultured on bovine bone slices and treated with ZOL and varying concentrations of BE. The results revealed that morphologically, the size of mature osteoclasts treated with BE and ZOL was significantly reduced compared with that of the control group (Figure 2B,D). It was intriguing that morphological irregularity and cytoskeleton retraction after BE treatment in the F‐actin ring formation experiments were consistent with the decreases in the number of bone resorption pits.

### 3.3 BE down‐regulated osteoclast‐specific gene expression

**FIGURE 3:**
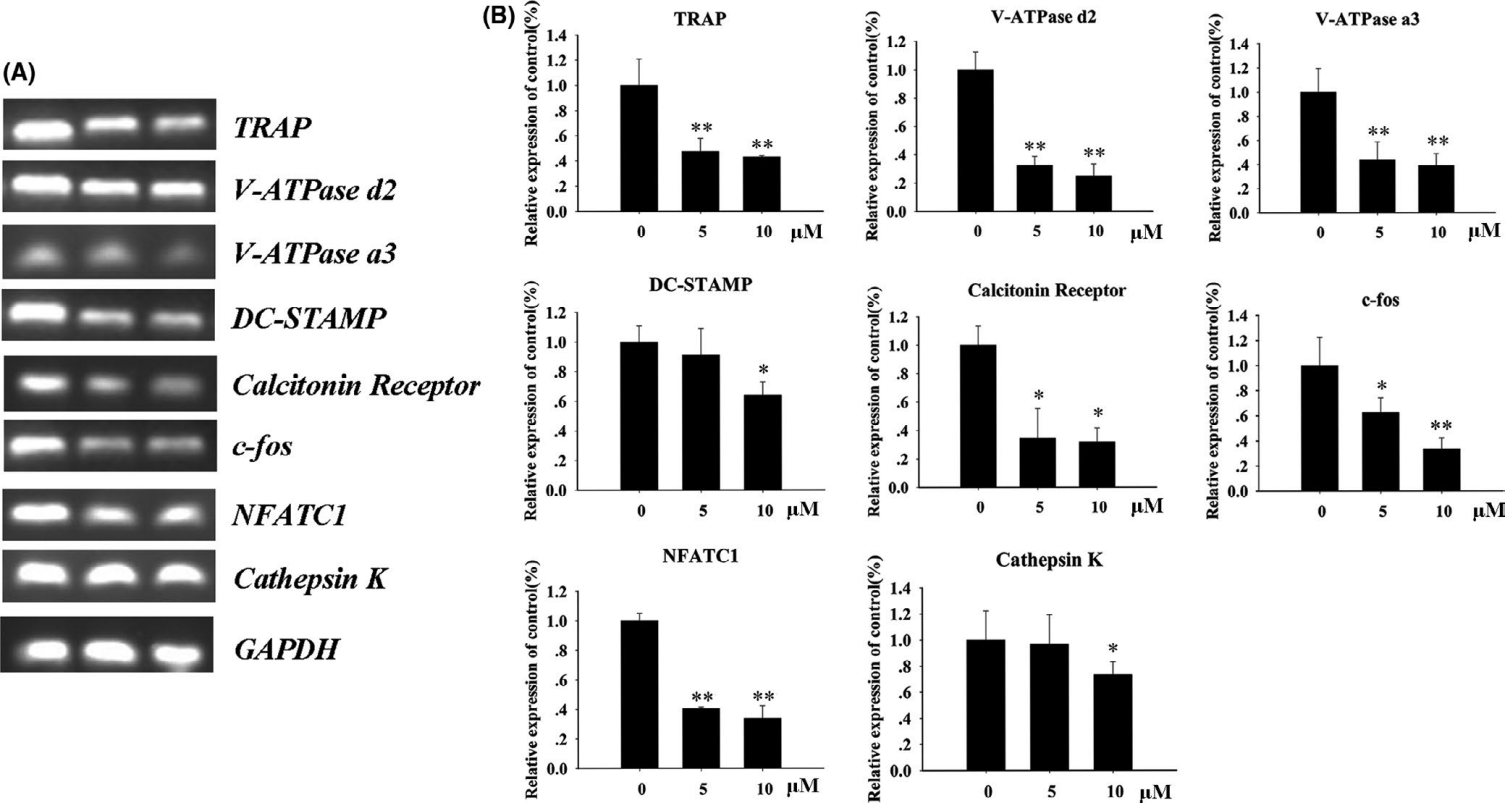
Bisphosphonate‐enoxacin (BE) downregulates RANKL‐induced osteoclast‐specific gene expression. (A, B) Bone marrow monocyte/macrophage cells (BMMs) were incubated in the presence of 30 ng/ml M‐CSF and 50 ng/ml RANKL with various concentrations of BE (0, 5 and 10 μM). Once mature osteoclasts were observed, total RNA was extracted and expression levels of indicated mRNAs were evaluated. TRAP, V‐ATPase d2, V‐ATPase a3, DC‐STAMP, Calcitonin Receptor, c‐fos, NFATC1 and Cathepsin K mRNA levels were quantified using quantitative PCR. All the experiments were carried out at least three times. The data are expressed as mean ± SD. *p < 0.05 and **p < 0.01 vs. the control group

It is known that RANKL can up‐regulate the expression of osteoclast‐related genes during osteoclast differentiation.^29^ Therefore, to confirm the suppressive effect of BE on osteoclast formation, we evaluated the effects of BE on the expression of osteoclast‐related genes TRAP, c‐fos, NFATc1, V‐ATPased2, V‐ATPasea3, CTR, DC‐STAMP and Cathepsin K. Compared with the control group, treatment with 5 and 10 μM BE inhibited expression of these genes in a dose‐dependent manner (Figure 3A,B). These findings confirmed that BE was able to downregulate osteoclast formation and osteoclast‐specific gene expression.

### 3.4 BE suppressed osteoclastogenesis through inhibiting the JNK signalling pathway

**FIGURE 4:**
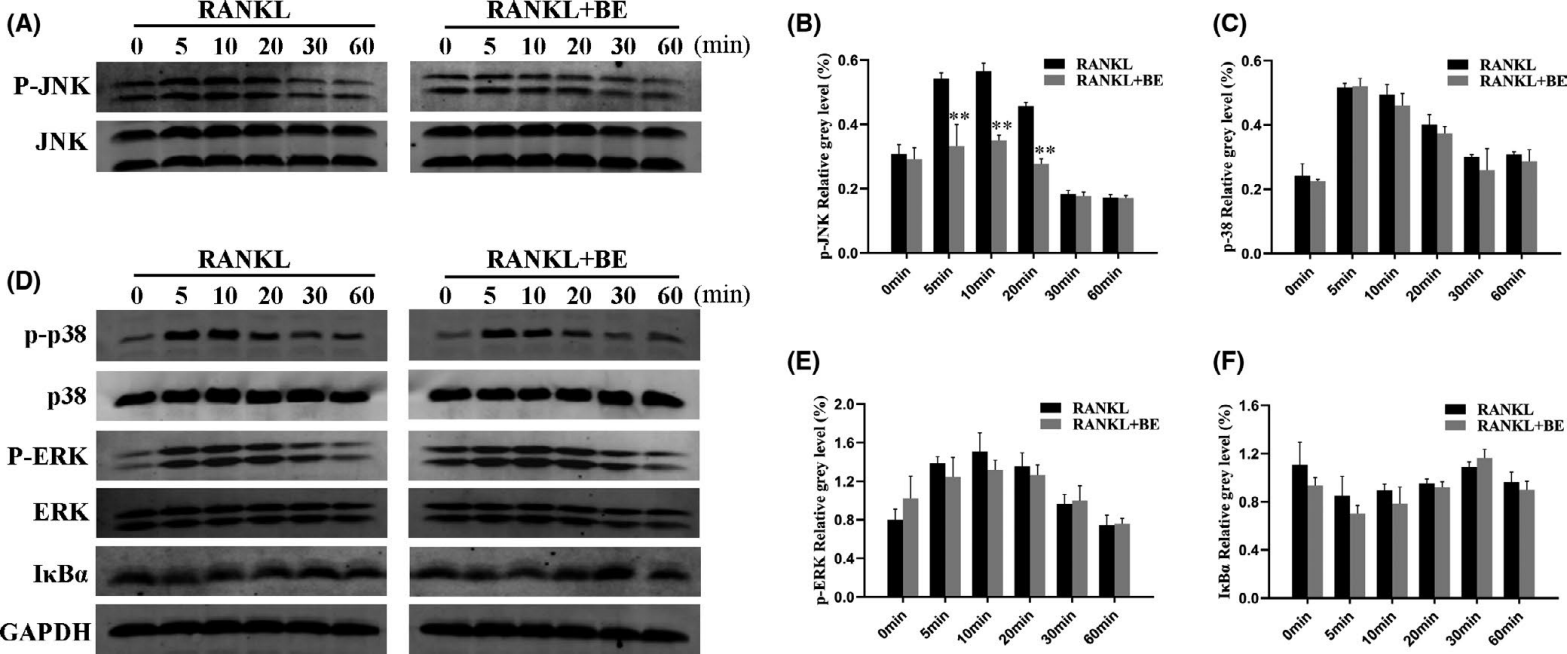
Bisphosphonate‐enoxacin (BE) suppresses RANKL‐induced JNK signalling pathways. (A) RAW264.7 cells were pre‐treated with or without 10 μM BE for 4 h and then stimulated with 50 ng/ml RANKL for 0, 5, 10, 20, 30 or 60 min. Total cell lysates were analysed by Western blotting using specific antibodies against phospho‐JNK, JNK and GAPDH. In the control group, JNK is rapidly phosphorylated within 5 min but greatly reduced in the presence of BE. (B) Band intensity corresponding to P‐JNK/JNK was quantitated and plotted using Image J software. (C–F) Western blotting was used to analyse protein expression levels of IκBα, phospho‐ERK, ERK, phospho‐p38 and p38. No differences were observed in the phosphorylation of the cascade between the control and BE‐treated groups. The findings were confirmed that no significant differences existed in IκBα/GAPDH, p‐ERK/ERK and p‐p38/p38 quantities. All the experiments were carried out at least three times. The data are expressed as mean ± SD. *p < 0.05 and **p < 0.01 vs. the control group

When RANKL specifically binds to the receptor RANK on the surface of progenitor cells, several intracellular signal transduction pathways in osteoclasts are initiated, including the two classical pathways NF‐κB pathway and MAPK pathway.^30^ JNK is a member of the MAPK family with a core effect of JNK phosphorylation being activated by the activation of kinases, which is the activation of downstream transcription factors and promotion of osteoclast differentiation. In the current study, we first investigated the effect of BE on the JNK signalling pathway. The results showed that in the control group, JNK phosphorylation was enhanced within 5–20 min after RANKL stimulation. However, after BE treatment, the phosphorylation of JNK was significantly reduced (Figure 4A,B). These results suggested that BE treatment inhibited JNK phosphorylation during osteoclast differentiation. We then used Western blotting to investigate other signal pathways closely related to osteoclast differentiation, including p38, ERK and NF‐κB signalling pathways. Interestingly, we found that when RAW264.7 cells were stimulated with RANKL, BE had no significant effect on signal transduction of the NF‐κB pathway as it was related to proteins IκBα, p38 or ERK, an observation further supported by the quantitative data (Figure 4C‐F). These data suggested that BE primarily suppressed osteoclast formation by inhibiting the JNK signal pathway, without affecting p38, ERK or NF‐κB.

### 3.5 BE prevented OVX‐induced bone loss in vivo by inhibiting osteoclast activity

**FIGURE 5:**
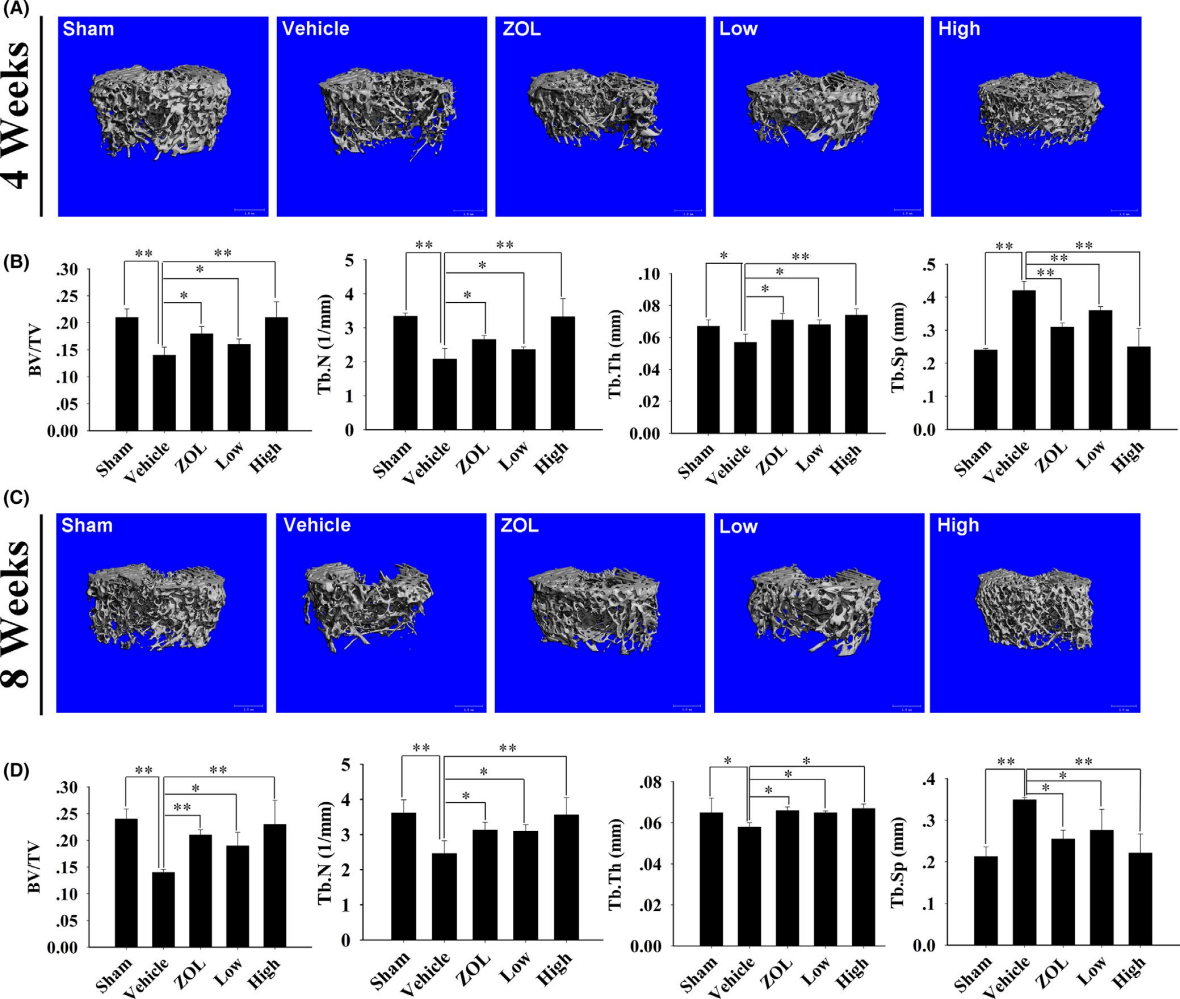
Bisphosphonate‐enoxacin (BE) prevents ovariectomized (OVX) rat bone loss *in vivo*. (A, C) Representative three‐dimensional (3D) reconstructed images of the right distal femurs from the five groups of rats at 4 and 8 weeks after the operation. (B, D) Micro‐CT analyses of bone volume to tissue volume (BV/TV; %), trabecular number (Tb.N,1/mm), trabecular thickness (Tb. Th, mm) and the trabecular separation (Tb. Sp, mm) for each sample in the region of interest. All the experiments were carried out at least three times. The data are expressed as mean ± SD. *p < 0.05 and **p < 0.01 vs. the vehicle group

To determine whether BE could prevent *in vivo* bone loss caused by oestrogen deficiency, we used the OVX rat model, which mimics postmenopausal osteoporosis. Micro‐CT scanning of the right distal femur of the five groups of rats 4 and 8 weeks post‐treatment revealed that the OVX groups experienced significant decreased trabecular bone volume compared with that of the sham control group, while bone loss in the ZOL‐treated group, low‐dose BE‐treated group and high‐dose BE‐treated group were significantly less compared to that of the OVX group (Figure 5A,C). This indicated that both BE and ZOL positively affected the skeletal structure of OVX rats Quantitative bone parameters further reflected that the BE‐treated group and ZOL‐treated group significantly increased Bv/Tv, Tb.N and Tb. Th, with decreased Tb. Sp (Figure 5B,D). Furthermore, the ZOL‐treated groups showed a trend of superior trabecular bone volume compared to that of the low‐dose BE‐treated group, although the differences between the two groups failed to reach statistical significance. Unexpectedly, we observed that the animals in the high‐dose BE‐treated group seemingly had a greater increase in trabecular bone volume compared to that in the ZOL‐treated group.

**FIGURE 6:**
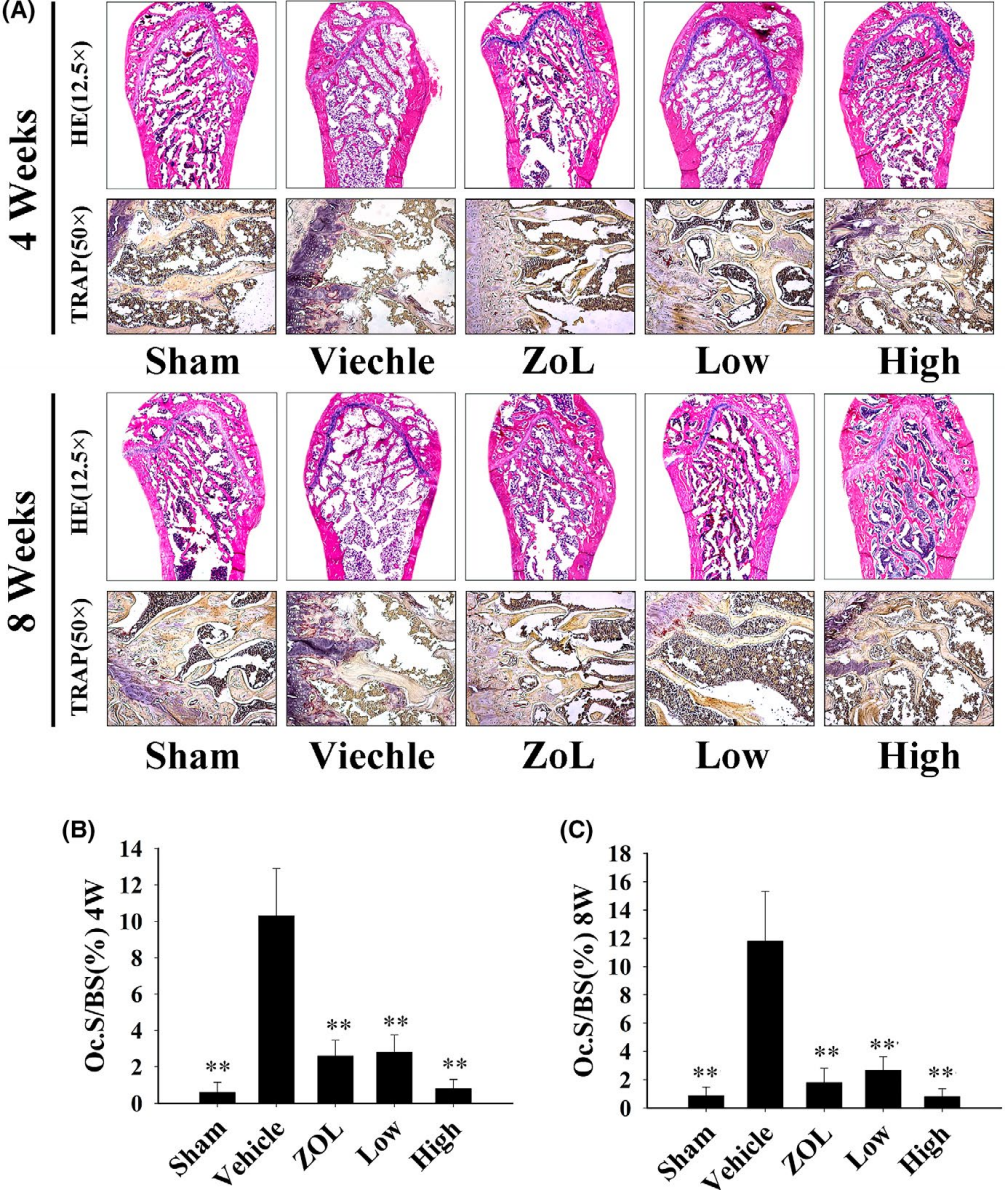
Bisphosphonate‐enoxacin (BE) ameliorates ovariectomized (OVX) rat bone loss based on histological and bone histomorphometric changes. (A) Haematoxylin and eosin (H&E) staining of representative sections of distal right femurs from each group of animals at 4 and 8 weeks after the operation. Representative images of tartrate‐resistant acid phosphatase (TRAP) staining are also shown. (B) Quantification of the osteoclast surface per BS (OC.S/BS) in each group at 4 weeks and (C) 8 weeks. All the experiments were carried out at least three times. The data are expressed as mean ± SD. *p < 0.05 and **p < 0.01 vs. the vehicle group

Considering that osteoclasts are the key effector cells for bone adsorption, we also analysed the number of TRAP‐positive multinucleated osteoclasts in rat femoral sections. Figure 6A–C shows that the number of osteoclasts after treatment with BE or ZOL was markedly reduced. Comparing the 4‐week‐treated and 8‐week‐treated groups, there appeared to be fewer TRAP‐positive cells in the 8‐week group compared to that in the 4‐week group.

**FIGURE 7:**
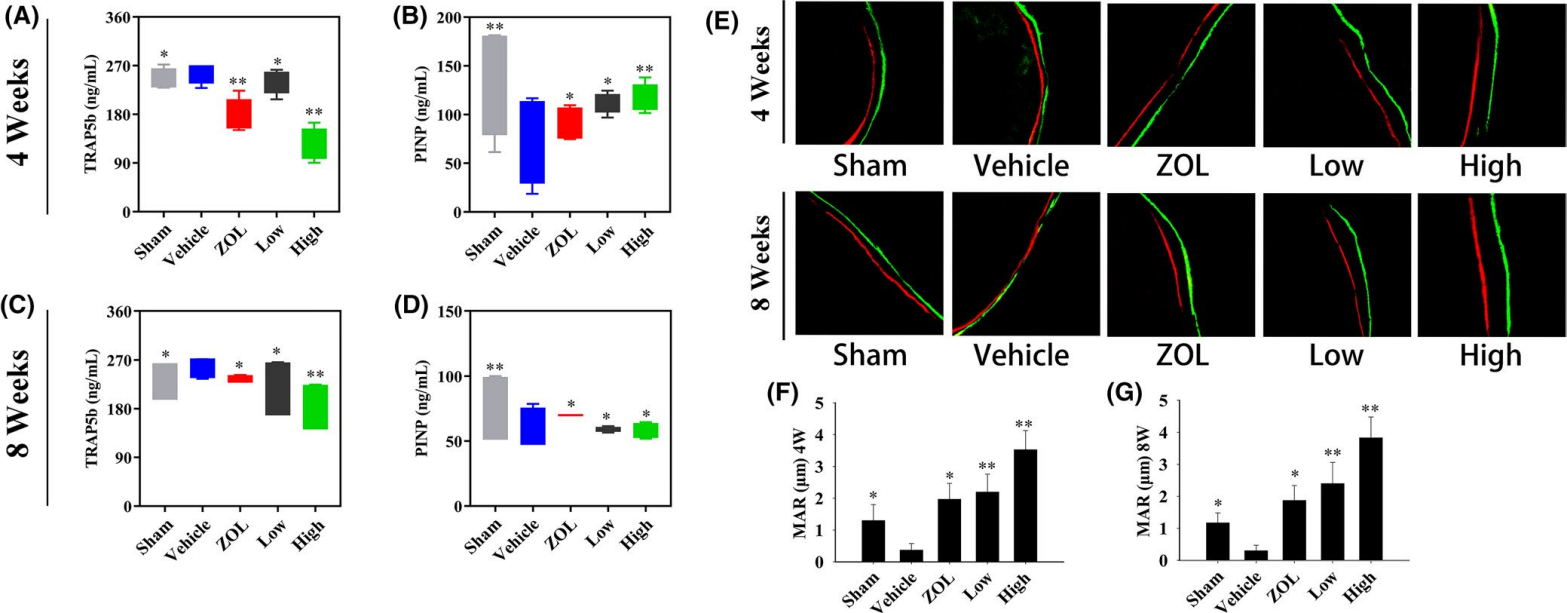
Evaluation of bone turnover markers and bone formation after treatment with bisphosphonate‐enoxacin (BE). (A–D) Serum levels of TRAP5b and PINP were examined at 4 and 8 weeks. (E–G) Calcein green and Alizarin Red S double labelling of distal left femurs at 4 and 8 weeks. Mineral apposition rate (MAR) is remarkably reduced in OVX rats and reversed by treatment with BE or zoledronate (ZOL). The BE appears to have superior efficacy compared to that of ZOL in terms of the significant restored MAR. All the experiments were carried out at least three times. The data are expressed as mean ± SD. *p < 0.05 and **p < 0.01 vs. the vehicle group

Next, we measured the level of TRAP5b released by osteoclasts into the serum, which is a well‐known marker of osteoclast activity and bone resorption. Figure 7A,C shows that TRAP5b activity in OVX rats was significantly decreased in the BE‐treated groups and ZOL‐treated group. Moreover, the effect of BE on TRAP5b levels showed a dose‐dependent inhibitory trend. A greater extent in the reduction of TRAP5b was observed in the high concentration BE‐treated group compared to that in the low concentration BE‐treated group.

Since PINP is a marker of bone formation,^31^ we also measured PINP levels in the serum. Figure 7B,D shows that both the high‐dose and low‐dose BE‐treated groups and the ZOL‐treated group had significantly higher levels of serum PINP compared to that in the untreated OVX group. Furthermore, PINP levels seemed to increase significantly in the BE‐treated groups compared with that in the ZOL‐treated group.

Fluorescent dyes injected in rats can specifically bind with mineralized tissues *in vivo* and auto‐fluoresce during the osteogenic repair process. Therefore, the growth rate of new bone tissue in the defect area can be analysed based on the distance between the two fluorescent bands. As shown in Figure 7E, we detected Calcein and Alizarin Red S fluorescence bands in each treatment group from both the 4‐wk and 8‐wk groups with the green fluorescence band representing the Calcein dye and the red fluorescence band representing the Alizarin Red S dye. We found that the distance between the Calcein green band and Alizarin Red S band was closer in the OVX group compared with that in the sham control group, indicating the rate of new bone formation was slower in the OVX group. Our findings also indicated that the distance between the two fluorescent bands in the BE‐treated group and the ZOL‐treated group was greater than that in the sham control group, which indicated that BE and ZOL effectively promoted new bone formation and significantly increased MAR (Figure 7E–G). It is intriguing that both the low‐dose and high‐dose of BE appeared to be superior in restoring bone loss compared to that of ZOL.

### 3.6 BE in vivo treatment appeared to be safe in major organs

**FIGURE 8:**
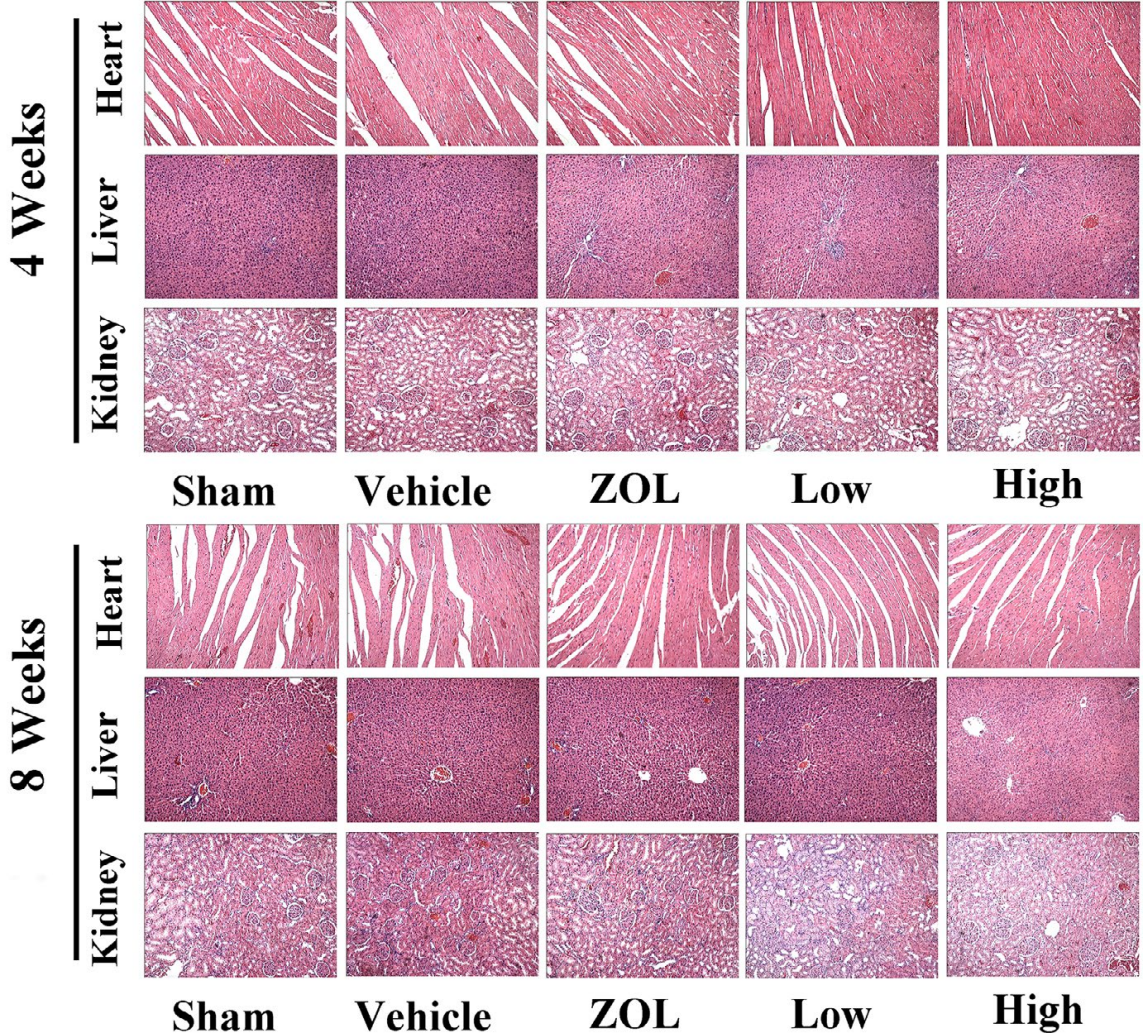
Safety of bisphosphonate‐enoxacin (BE) use *in vivo* in major organs. Histological morphology of major organs including the heart, liver and kidney was stained using H&E at 4 and 8 weeks. All the treated groups showed similar histological morphology to that of the sham control group with clear cell structures, normal nuclei morphology and no abnormal histological manifestations such as shrinkage, oedema and necrosis being found

To evaluate the safety of BE and ZOL *in vivo*, we carefully monitored the condition of the rats at 4 and 8 weeks after intraperitoneal injection of BE and ZOL. No abnormal behaviour was exhibited by animals in any of the groups of rats. This indicated that BE and ZOL were safe and did not induce any obvious side effects in the rats. Tissue sections of major organs including the heart, liver and kidney were stained with H&E 4 and 8 weeks after BE treatment and histopathologically evaluated. All the treated groups showed similar histological morphology to that of the sham control group with clear cell structures, normal nuclei morphology and no abnormal histological manifestations such as shrinkage, oedema and necrosis being found (Figure 8). Additionally, as shown in Table 1, cardiac function (CK) and liver function (AST) demonstrated only slight differences among the different groups and the rats showed no obvious toxicity after short (4 weeks) and relatively long (8 weeks) periods of treatment with BE and ZOL. However, renal function (BUN) showed abnormalities for 8 weeks in the ZOL treatment group, and the difference was statistically significant, while the BE treatment group had no effect. The results of these *in vivo* experiments suggested that BE was even safer than ZOL on the occurrence of side effects associated with long‐term treatment.

**TABLE 1:** The indicator of the effect of bisphosphonate‐enoxacin (BE) on heart, liver and kidney function

	Sham	Vehicle	ZOL	Low	High
4‐week					
CK(U/L)	1267 ± 284	1357 ± 251	1193 ± 225	1207 ± 225	1292 ± 243
AST(U/L)	98 ± 8	97 ± 12	108 ± 46	104 ± 16	84 ± 8
BUN(mmol/L)	7.2 ± 1.6	7.1 ± 1.7	7.0 ± 0.5	7.3 ± 1.0	7.8 ± 1.0
8‐week					
CK(U/L)	1622 ± 167	1630 ± 338	1703 ± 441	1873 ± 355	1942 ± 188
AST(U/L)	116 ± 11	105 ± 8	118 ± 4	114 ± 27	107 ± 16
BUN(mmol/L)	8.0 ± 0.3	7.5 ± 0.6	10.8 ± 1.0*	9.3 ± 0.9	8.4 ± 1.1

Note

The data are expressed as mean ± SD. *p < 0.05 vs. the sham group.

Abbreviation: CK, Cardiac function; AST, liver function; BUN, renal function.

## 4 DISCUSSION

Osteoporosis is a challenging diagnosis in the initial stages of the disease and is rarely diagnosed before a fracture occurs.^32^ Osteoporotic fractures severely affect public health and increase medical costs, thereby being a significant economic burden on society. In terms of treatment of osteoporosis diseases, clinical choices of drugs are more or less limited. Traditional herbs and herbal extracts may exhibit a certain level of effect as anti‐osteoporosis agents,^33^ but remain inadequate. Furthermore, due to the relatively low vascularization and physical barriers that impact the ability of a drug to penetrate tissues, higher doses are often required, which may lead to unwanted systemic effects or side effects resulting in high‐dose medication being limited or excluded for their use in the treating bone diseases.^34^, ^35^ Fortunately, we demonstrated that BE developed by the Holliday group^21^ as a bone‐targeting derivative of BP was highly successful in improving cortical bone mass and enhancing bone biomechanical properties, as confirmed by our previous research.^36^ However, the specific mechanism involved in BE preventing bone loss and its safety remained to be elucidated.

In our current study, we first performed a CCK‐8 assay to determine the effects of BE and ZOL on osteoclast precursor proliferation. Doses of 5 and 10 μM BE and 2.5 μM ZOL were selected for our *in vitro* studies aimed to exclude BE and ZOL induction of cytotoxic effects. We found that BE had fewer side effects on the proliferation of osteoclast precursors and significantly inhibited the expression of RANK, thereby inducing osteoclast precursors to differentiate into osteoclasts. Therefore, RANKL‐related downstream signalling pathways may be potential targets for regulating osteoclast formation and bone resorption. RANKL is a member of the tumour necrosis factor (TNF) receptor family, which recruits TRAF in conjunction with RANK and leads to the activation of the NF‐κB and MAPK pathways.^37^, ^38^ NF‐κB exists in the cytoplasm of cells and binds to IκBα inhibitor protein in an inactive form. Upon the binding of RANKL and RANK, IκBα is phosphorylated by the IκB kinase complex, resulting in IκBα ubiquitination and degradation followed by the activation and translocation of NF‐κB to the nucleus to initiate transcription.^39^ Our current results indicated that BE did not effect IκBα phosphorylation.

MAPK signalling pathways, including JNK, ERK and p38, are important regulatory pathways during osteoclast formation.^29^ Our study found that when RANKL was added, the JNK, ERK and p38 signalling pathways were activated. However, when RANKL and BE were added at the same time, BE did not change the RANKL‐induced activation of ERK and p38, but did inhibit phosphorylation of the JNK pathway. Previous studies have revealed that as an upstream target of AP‐1 the JNK signalling pathway plays an equally important role in RANKL‐induced osteoclast formation.^40^ Thus, the decrease in JNK activity may also interfere to some extent with RANKL‐induced osteoclast formation. Taken together, our findings show that BE was able to inhibit RANKL‐induced JNK signal transduction without affecting p38, ERK, or NF‐κB pathways.

NFATc1 regulates osteoclast differentiation with the expression of function‐related genes, such as TRAP, c‐fos, NFATc1, V‐ATPased2, V‐ATPasea3, CTR, DC‐STAMP and Cathepsin K, playing important roles in osteoclast differentiation and bone resorption.^29^, ^41^ The results of our current study show that BE inhibited the expression of NFATc1 at the mRNA level and down‐regulated the expression of the above‐mentioned genes, suggesting that BE affected both the expression of NFATc1 and the expression of its downstream genes, which also reasonably explains the effect of BE inhibition of osteoclastogenesis. Furthermore, ruffled membrane, also known as F‐actin ring, is a prominent feature of polarized osteoclasts, and a well‐polarized F‐actin ring is a prerequisite for effective bone resorption.^42^ In our study, BE impaired RANKL‐inducing osteoclast formation of F‐actin and inhibited osteoclast‐mediated bone resorption in a concentration‐dependent manner. Previous studies have shown that two proteases, DC‐STAMP and cathepsin K, are expressed by osteoclasts and play a key role in osteoclast resorption activity.^43^, ^44^ Our research showed that after BMMs were treated with BE, expression of DC‐STAMP and cathepsin K mRNAs was significantly reduced in concert with bone resorption pits being reduced, suggesting that BE inhibited the expression of DC‐STAMP and cathepsin K leading to the inhibition of osteoclast‐mediated bone resorption. Thus, we conclude that BE affected not only the differentiation of osteoclasts, but also the bone resorption function of osteoclasts.

We also used an *in vivo* osteoporosis model induced by oestrogen deficiency in ovariectomized rats, which causes reduced bone density and bone microstructure changes similar to those observed in humans.^45^ We found that the BE‐treated group and ZOL‐treated group exhibited remarkably reduced bone loss in OVX rats. H&E staining and micro‐CT analysis further confirmed that BE and ZOL prevented OVX‐induced bone loss by inhibiting osteoclastogenesis. TRAP staining and histomorphometric analysis showed that the number of TRAP‐positive multinucleated osteoclasts proximal to the trabecular bone of the distal femur was significantly decreased after intraperitoneal injection of either BE or ZOL. Interestingly, we found that the high‐dose BE‐treated group seemed to induce better biomechanical properties with respect to preventing and treating bone loss compared to that of the ZOL‐treated group. Additionally, serum levels of TRAP5b, which are up‐regulated after OVX, were down‐regulated after treatment with BE. It was clear that BE reduced bone loss in OVX rats by promoting osteoclasts. Additionally, serum levels of PINP were reduced after OVX, but PNIP activity was elevated after BE treatment, suggesting that BE promoted bone formation in OVX rats. The fluorescence double‐label experiment further confirmed the increase in bone matrix mineralization.

Although enoxacin has been previously reported to inhibit osteoclast bone resorption^18^, the local concentrations required to inhibit osteoclasts in the skeletal microenvironment required the use of large systemic doses, which resulted in side effects that were catastrophic. Moreover, the high doses of enoxacin usually killed the commensal bacteria and may also for the evolution of resistant strains. Therefore, it is difficult to imagine enoxacin being used to treat bone diseases such as osteoporosis. Fortunately, zoledronate is quickly located to bone where it accumulates, allowing relatively low systemic doses resulting in therapeutic doses in the bones. Thus, combining the best characteristics of both these agents as a bone‐targeting anti‐osteoclast resorption agent seemed perfectly suitable for the treatment of osteoporosis, and is precisely what our current study confirmed. Thus, BE is more effective than zoledronate as an inhibitor of bone resorption, presumably because of the increased local concentration associated with the bone surface, which could be mobilized as osteoclasts began to resorb the bone. Another possibility is that V‐ATPases are known to be integrated into various metabolic and physiological pathways.^46^ BE may disrupt the targeting of V‐ATPases thus leading to the release of extracellular vesicles causing alterations in microRNA levels.^47^ Subsequently, stimulation of microRNAs, rescue of p53^48^ or stimulation of the JNK pathway may produce a variety of cellular effects that are distinct from BP action on bone.

In our current work, we observed that BE had an anti‐osteoclastic effect, which indicates that it may have enormous potential for wide application in humans as an anti‐osteoporosis treatment and even possibly as an anti‐osteoclast‐related osteolytic disease therapy. Certainly, there are several reasons for this assumption. The primary reason for the minimal or non‐existent toxicity seen in the rats after treatment with BE during the time frame of the experiments is that the study used systemic injection of BE instead of oral consumption. The second reason is that BE is well tolerated and no abnormal behaviour or other discomfort occurred in the mice. Furthermore, we failed to observe adverse effects of BE on the proliferation of BMMs at the safe concentrations used *in vitro*. Moreover, our observed success *in vivo* in part was based on the fact that treatment with BE prevented bone loss in OVX rats, but also because the histopathological analysis and a series of biochemical indicators tests showed that BE did not induce any noticeable toxic effects on major organs, including the heart, liver and kidney. Thus, our study not only considered the efficacy of BE, but also systematically evaluated the safety of BE, both *in vitro* and *in vivo*.

Overall, our findings are novel and contribute towards the biological applications and clinical transformation of BE as a treatment for osteoporosis and other osteolytic diseases. However, there are some limitations to our studies. The pathological process of osteoporosis involves both osteoclastic bone resorption and osteoblastic bone formation. While our present study confirmed that BE significantly inhibited osteoclast differentiation *in vitro* and *in vivo* and reduced OVX‐induced bone loss, the effects of BE on bone formation and its exact mechanism still need to be fully addressed. Additionally, although we demonstrated that BE affected osteoclastogenesis by inhibiting the expression of NFATc1 and other osteoclast‐related markers by targeting the JNK‐MAPKs signalling pathway, the specific kinases involved need to be resolved.

## 5 CONCLUSIONS

In summary, the results of our present study indicated that BE inhibited osteoclast differentiation and functioned both *in vitro* and *in vivo*. We also found that BE inhibited the RANK‐induced JNK signalling pathway and ultimately attenuated expression of the transcription factor NFATc1, resulting in the downregulation of osteoclast‐specific genes and thereby inhibiting osteoclast formation. Moreover, to our knowledge, this is the first report of BE preventing OVX‐induced bone loss under the premise that it highly safe both *in vivo* and *in vitro* and also provided the first real insight into the relevant mechanisms involved. We also demonstrated that BE was equal or superior at preventing and treating bone loss compared with zoledronate, which is widely used in anti‐osteoporosis treatment. Furthermore, we showed that BE was even safer than zoledronate and may prove to be a feasible approach to reduce the occurrence of side effects associated with long‐term treatment. Based on these findings, we believe that BE is a novel bone‐targeting derivative with unique advantages and may provide a potentially new strategy in the treatment of osteoporosis.

## CONFLICT OF INTEREST

The authors declare no competing financial interests.

## AUTHOR CONTRIBUTION

**Qiang Xu:** Data curation (equal); Formal analysis (equal); Investigation (equal). **Xiaofeng Li:** Data curation (equal); Methodology (equal); Project administration (equal). **Fengbo Mo:** Project administration (equal); Resources (equal); Writing – review and editing (equal). **Huaen Xu:** Data curation (equal); Software (equal). **Yuan Liu:** Supervision (equal); Writing – original draft (equal). **Qi Lai:** Data curation (equal); Validation (equal). **Bin Zhang:** Visualization (equal); Writing – original draft (equal). **Min Dai:** Conceptualization (equal); Funding acquisition (equal). **Xuqiang Liu:** Conceptualization (equal); Funding acquisition (equal). **Ping Zhan:** Data curation (equal); Writing – review and editing (equal).

## Data Availability

The data that support the findings of this study are available from the corresponding author upon reasonable request.
